# Efficacy of Lactose-Free Milk in Treating Acute Gastroenteritis in Vietnamese Children: A Randomized Controlled Trial

**DOI:** 10.7759/cureus.61178

**Published:** 2024-05-27

**Authors:** Rang N Nguyen, Nghia Q Bui, Diep N Thai

**Affiliations:** 1 Pediatrics, Can Tho University of Medicine and Pharmacy, Can Tho, VNM

**Keywords:** children, diarrhea, acute gastroenteritis, lactose-containing milk, lactose-free milk

## Abstract

Background:Low lactase levels in Asian children appear to be genetically determined or rotavirus-induced gastroenteritis. Consuming lactose-free formula in children with acute gastroenteritis may shorten diarrhea's duration and increase weight gain. This study aims to determine whether lactose-free milk will change the duration of diarrhea and weight gain in Vietnamese children aged 2-24 months with acute gastroenteritis.

Materials and methods: A randomized control trial was performed on 66 children under 24 months of age with acute gastroenteritis at the Gastroenterology Department of Can Tho Children’s Hospital. In adjunction to oral rehydration solution, they received either a lactose-free formula (n=33) or a lactose-containing formula (n=33).

Outcome measures: Diarrhea duration, weight gain, treatment failure, and days of hospitalization were all studied.

Results: A total of 66 children participated in this trial, with a mean age of 13.4 ± 5.1 months, and 38 participants (57.6%) were male. There were no significant differences between the lactose-free formula group and the lactose-containing formula group in the duration of diarrhea (2.2±0.8 days versus 2.4±0.9 days; P=0.321), percentage of weight gain (1.96 [IQR:1.35-2.36] percent vs. 2.29 [IQR:1.81-2.40] percent; P=0.131), treatment failure rate (33.3% vs. 36.4%; P= 0.796), and days of hospitalization (5.8±1.7 vs. 6.5±2.5 days; P=0.158).

Conclusions: It may not be necessary to use lactose-free milk routinely in Vietnamese children under 24 months with acute gastroenteritis as the duration of diarrhea, weight change, treatment failure rates, and hospital stay are similar to those of children fed lactose-containing milk.

## Introduction

Childhood diarrhea remains a serious global public health issue, with an estimated 1.7 billion infections and 72% of deaths caused by diarrhea occurring in the first two years of life [1]. There are more than 115 diarrhea episodes per 1000 child-years in Vietnam among children under five, and most cases occur in children under two years of age [2,3].

A number of studies show that Asians have a higher prevalence of lactose malabsorption than people of European descendants. Up to 98% of Vietnamese people suffer from lactose malabsorption [4]. Furthermore, rotavirus is responsible for most diarrhea cases in Vietnamese children, which results in the loss of lactase-producing epithelial cells from the villi tips [5]. Therefore, it is hypothesized that providing lactose-free milk to Vietnamese children with acute gastroenteritis may reduce the duration of diarrhea and reduce weight loss during acute diarrhea episodes requiring hospitalization.

Several studies conducted in Asian countries found that children with acute gastroenteritis who consumed lactose-free milk had shorter diarrheal duration and recovered weight faster [6-10]. A meta-analysis by MacGillivray et al. found that lactose-free products may reduce the duration of diarrhea by an average of about 17.7 hours (95% CI: 10.2-25.3 hours) [11]. In contrast, a number of studies reported that feeding children lactose-free milk did not appear to change the duration of diarrhea [12-15]. Therefore, the use of lactose-free milk for children with acute gastroenteritis remains controversial.

The purpose of this study is to determine whether lactose-free milk will change the duration of diarrhea and weight gain in Vietnamese children with acute gastroenteritis.

## Materials and methods

Study design

A randomized controlled open-label parallel-arm clinical trial, with allocation 1:1, was conducted to investigate whether lactose-free milk will shorten the duration of diarrhea and change the weight gain in children with acute gastroenteritis when compared with lactose-containing milk.

Setting

This study was conducted at the Gastroenterology Department of Can Tho Children’s Hospital from December 2022 to December 2023.

Sample size

We hypothesize that the lactose-free formula will shorten the diarrhea duration by an average of 18 hours (±24 hours) as compared with the lactose-containing formula [10,11]. To detect this difference between the two groups with a power level of 80% and 5% type I error, the sample size calculated was 33 subjects in each group. Considering a 10% attrition rate, the sample size was calculated as 37 in each group. The final sample size was 74.

Inclusion and exclusion criteria

Infants and children between the ages of 2 and 24 months hospitalized with acute gastroenteritis feeding on artificial milk formula were eligible to participate.

Acute diarrhea is defined as the passage of three or more loose stools in 24 hours, for at least 24 hours, and not exceeding two weeks from presentation, without blood in the stool [16]. Children with any of the following conditions will be excluded: use of antibiotics within three days before admission, breastfeeding, sepsis, chronic diarrhea, severe dehydration (loss of over 10% of total body water), milk allergy, necrotizing enterocolitis, intussusception, consumption of lactose-free milk, immune deficiency, and chronic illness.

Randomization

Eligible infants will be randomly allocated to one of two parallel groups (intervention and control, 1:1 ratio) by using the RAND() function in Excel (Microsoft Excel, Microsoft® Corp., Redmond, WA). The random sequence will be generated by an independent biostatistician. Allocation concealment will be done to ensure that group assignments of the participants are revealed only after assessing that the inclusion/exclusion criteria are verified and consent obtained. A set of sequentially numbered opaque sealed envelopes will be prepared with the allocation group, as per the randomization list specified inside.

Control Group

Children will receive standard medical care, including rehydration with oral rehydrating solutions (ORS), zinc supplementation, and symptomatic treatment. According to the research team, the family will receive lactose-containing milk (100 ml/kg/day) instructions along with their antidiarrheal dietary instructions. Additional treatment by the primary physician, including dietary changes (whether milk or solid foods), will be respected. If a patient in the control group does not improve on the lactose-containing milk, he/she will be referred to his/her primary physician for further treatment, including switching to a lactose-free formula. All dietary changes or medication intake during the study period will be recorded as part of data collection.

Intervention Group

Children will receive standard medical care, including rehydration with oral rehydrating solutions, as per their physicians’ recommendations. The dietary instructions will be provided to the family by the research team and will consist of the same antidiarrhea diet prescribed to the control group, in addition to the lactose-free formula (Frisolac LF®) 100 ml/kg/day. Any additional treatment by the primary physician, including dietary changes (whether milk or solid foods), will be respected. If a child in the intervention group does not improve on the lactose-free formula, he/she will be referred to his/her primary physician for further treatment, including switching to any other lactose-free or lactose-containing formula.

Operational definitions

Operational definitions are as follows: (i) the duration of diarrhea was defined as the number of days with three or more loose or watery stools, from randomization until the day of the last diarrhoeic stool passed. (ii) Percentage of weight gain was calculated as (weight at the time of discharge - admission weight) ×100/weight at the time of discharge. (iii) Treatment failure was defined as a clinical requirement for intravenous infusion after rehydration or prolonged diarrhea (>7 days). (iv) Malnutrition was defined when the weight for age Z-score (WAZ) was less than −2 standard deviation (SD) by using the WHO Anthro 3.0 software [17]. (v) The clinical severity of gastroenteritis was scored according to the Vesikari 0-20 point numerical scores [18]. (vi) The reducing sugars in the stool were detected by using Benedict’s test. (vii) Rotavirus in stool samples was detected by a real-time PCR using a CFX96 TouchTM (Bio-Rad Laboratories, Hercules, CA, USA), conducted at the Nam Khoa Biotek Company, Ho Chi Minh City, Vietnam, in accordance with ISO 9001:2015.

Outcomes

The primary outcome measurement in this study was the duration of diarrhea (days). The secondary outcomes were the percentage of weight gain, the treatment failure rate and the duration of hospitalization.

Ethical approval

The study was approved by the Scientific and Ethical Committee of Can Tho University of Medicine and Pharmacy and Can Tho Children’s Hospital. The study was registered at ClinicalTrials.gov (NCT05634603). Informed consent was obtained from the parents or care-givers of the participants after giving them a full explanation of the purpose of the study.

Statistical analysis

Categorical variables were expressed as numbers and percentages; continuous variables were expressed as mean and SD, or median and interquartile range, when appropriate. For categorical variables, the Chi-square test was used or Fisher’s exact when the expected values in any of the cells of a contingency table were below 5. For continuous variables, the Student's t-test or the Mann-Whitney-U test was used, depending on the validity of the normality assumption. Analyzes were performed with SPSS software (version 26.0). All P-values are two-sided, with a P-value of less than 0.05 was considered to indicate statistical significance.

## Results

**Figure 1:**
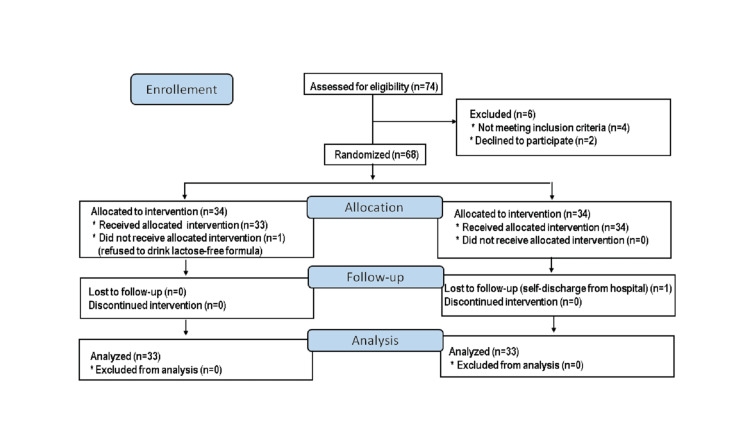
The CONSORT flow diagram of the study CONSORT: consolidated standards of reporting trials

A total of 74 patients with acute gastroenteritis on admission were screened for inclusion, of whom 68 were randomized and 66 (33 patients in each group) were included in the per-protocol analysis (Figure 1).

The mean age of 66 children was 13.4 ± 5.1 months, with 38/66 (57.6%) of whom were male. The demography, clinical, and paraclinical characteristics of the patients at baseline in the two groups are presented in Table 1.

**Table 1:** The baseline characteristics of diarrheal children at the enrolment *p-value < 0.05 was considered significant; ^a^Chi-squared/Fisher’s exact test, ^b^Student’s T-test/Mann–Whitney U-test; SD: standard deviation, IQR: interquartile range

	Lactose-free formula (n=33)	Control (n=33)	p-value
Male gender, number (%)	19 (57.6%)	19 (57.6%)	1.000^a^
Age, mean ± SD, months	12.7± 5.1	14.1± 5.1	0.271^b^
Body weight at admission, mean ± SD, kg	9.1 ± 1.5	9.7 ± 1.9	0.140^b^
Body temperature at admission, mean ± SD, °C	38.1± 0.8	38.3 ± 0.8	0.383^b^
Duration of diarrhoea before admission, mean ± SD, days	3.45 ± 0.93	3.52 ± 1.34	0.833^b^
Malnutrition, number (%)	0 (0%)	1 (3.0%)	1.000^a^
Maximum frequency of stools per day before admission, median (IQR)	7 (5–10)	7 (5.5–7)	0.928^b^
Maximum number of vomiting per day before admission, median (IQR)	4 (2–6)	3 (0.5–5.5)	0.297^b^
Grade of dehydration, number (%)			
Mild	27 (81.8%)	29 (87.9%)	
Moderate	6 (18.2%)	4 (12.1%)	0.492^a ^
Vesikari score, mean ± SD	12.15 ± 2.04	11.61 ± 2.46	0.331^b^
Serum electrolytes			
Sodium, mean ± SD, mmol/L	134.4 ± 5.8	134.9 ± 3.1	0.739^b^
Potassium, mean ± SD, mmol/L	3.8 ± 0.5	3.8 ± 0.6	0.798^b^
Chloride, mean ± SD, mmol/L	104.9 ± 5.8	102.4 ± 5.3	0.212^b^
Use of probiotics, number (%)	30 (90.9%)	31 (93.9%)	0.643^a^
Stool rotavirus: positive, number (%)	15 (45.4%)	14 (42.4%)	0.804^a^
Reducing substances: positive, number (%)	5 (15.2%)	8 (24.2%)	0.353^a^

Overall, there were no significant differences in gender, age, body weight at admission, body temperature, grade of dehydration, the Vesikari severity score, or abnormal electrolytes (sodium, potassium, or chloride) between the two groups. Also, the duration of diarrhea, maximum number of stools, and maximum number of bowel movements per day before admission were similar between the two groups. Reducing substances were detected at 15.2% in the lactose-free formula group and at 24.2% in the control group, respectively (p=0.353). Rotavirus was detected at 15/33 (45.4%) in the lactose-free formula group and at 14/33 (42.4%) in the control group, respectively (p=0.804). The number of cases using probiotics (Saccharomyces boulardii) in the lactose-free formula group and the control group were 30/33 (90.9%) and 31/33 (93.9%), respectively (p=0.643) (Table 1).

The mean duration of diarrhea in the lactose-free formula group was shorter (2.2 ± 0.8 days) as compared to those in the control group (2.4 ± 0.9 days), but there was no difference between the two groups (p=0.321).

**Table 2:** The main and secondary outcomes in lactose-free formula and control groups *p-value < 0.05 was considered significant; ^a^Chi-squared/Fisher’s exact test, ^b^Student’s T-test/Mann-Whitney U-test; SD: standard deviation, IQR: interquartile range

Main and secondary outcomes	Lactose-free formula (n=33)	Control (n=33)	p- value
Duration of diarrhea, mean ± SD, days	2.2 ± 0.8	2.4 ± 0.9	0.321^b^
Duration of hospitalization, mean ± SD, days	5.8 ± 1.7	6.5 ± 2.5	0.158^ b^
Treatment failure rate, number (%)	11 (33.3%)	12 (36.4%)	0.796^ a^
Body weight at discharge, mean ± SD, kg	9.3 ± 1.4	9.9 ± 1.9	0.107^ b^
Weight gain, median (IQR), %	1.96 (1.35–2.36)	2.29 (1.81–2.40)	0.131^ b^

The mean duration of hospitalization (5.8±1.7 vs. 6.5±2.5 days) was not statistically different between the two groups (p=0.158). Body weight significantly increased in both the lactose-free formula (9.1±1.5 to 9.3±1.4; P<0.001) and control groups (9.7±1.9 to 9.9±1.9 [CI 95%: 11 to 65 g]; p<0.001) at the time of hospital discharge, but there was no difference between the two groups in weight change (17±15 vs. 23 ±18 g, p=0.179). Children in the treatment group had a median (IQR) weight change of 1.96% (1.35-2.36%), which was not statistically different from the control group (2.29% [1.81-2.40%]; p=0.131). The treatment failure rate (33.3% versus 36.4%; p=0.796) was not different between the two groups (Table 2).

There were no allergies or other adverse effects observed in children who consumed either lactose-free or lactose-containing formula.

## Discussion

In this randomized controlled study, it was found that there was no difference in diarrhea duration between the lactose-free formula (2.2 ± 0.8 days) and the lactose-containing formula (2.4 ± 0.9 days; p>0.05) among Vietnamese children with acute gastroenteritis. Also, the duration of hospital stays was no different between the two groups. It is possible that mild dehydration and the well-nourished nature of our patients contributed to the short duration of diarrhea in both groups. Furthermore, we noted that only 15% of children in the lactose-free formula group were suspected of having lactose intolerance. It was consistent with several studies on Asian people, which indicated that lactose intolerance is almost nonexistent in children under two years of age, and it seems to appear after three years of age and gradually increases with age [19-21]. Therefore, the children who were fed lactose-free milk did not shorten the days of diarrhea in our study.

Several previous studies supported our findings. In a study on Indonesian children aged under two suffering from acute gastroenteritis and mild dehydration, Lestari et al. found that children who were fed with lactose-free formula had diarrhea lasting the same duration as those who were fed with lactose-containing formula [12].

Similarly, Lozano et al. found that Colombian children aged one to 24 months receiving lactose-free formula did not have shorter diarrhea symptoms when compared to those receiving lactose-containing milk [13]. Among Indian children aged under two suffering from mild acute gastroenteritis, Bahn et al. suggested that a cow’s milk-based formula is well tolerated after initial rehydration with oral rehydrating solutions, and the infants who were fed with cow’s milk showed significantly higher energy intake and weight gain than those who were fed with a milk-free formula [14]. In a meta-analysis, Brown et al. found that children who received lactose-containing diets during acute diarrhea with severe dehydration were twice as likely to have treatment failure as those who received a lactose-free diet (22% versus 12%, respectively; P<0.001). However, the treatment failure rate in the lactose group was similar to that in the lactose-free group in diarrheal children with no or mild dehydration. So the authors concluded that most children with acute gastroenteritis with mild dehydration can be successfully treated with oral rehydration therapy and undiluted milk formula, and the routine use of lactose-free formula is not necessary [22].

In contrast to our study, many studies from Asian countries found that children aged under two who consumed lactose-free milk had a shorter duration of diarrhea than those who consumed lactose-containing milk [7-10]. Particularly, Al-Dulaimy et al. reported that children under 12 months of age received lactose-free milk responses better than those who received lactose-containing milk [23]. A meta-analysis consisting of 33 trials, performed by MacGillivray et al., showed that diarrhea may last less than 17.7 hours (95% CI: 10.2-25.3 hours) in children fed with lactose-free milk [11]. Thus, lactose-free milk is still controversial for infants and children with acute diarrhea who are fed artificial milk. Using lactose-free milk appears to reduce the duration of diarrhea in diarrheal children with severe dehydration or those who are under 12 months of age [7,10,22,23]

Due to the short duration of diarrhea and the short duration of the hospital stay in both groups of this study, lactose-free formula did not significantly affect weight change during treatment for diarrhea. This observation is also in line with many previous studies [6,9,13,24]. Whereas a study conducted in Thailand revealed that children under two years who were fed lactose-free milk gained more weight after 24 hours of intervention, but no difference was seen after two to seven days [7].

In our study, the treatment failure requiring unscheduled intravenous fluid therapy was not significantly different between the lactose-free formula and the control group. In contrast, Simakachorn et al. reported that unscheduled intravenous fluid therapy could be increased by twice in the lactose-containing formula group [7]. Similarly, Fayad et al. showed that treatment with a soy-based formula with lactose was associated with a higher rate of treatment failure; the number of cases requiring intravenous fluids increased three times compared to the lactose-free group [25].

The strength of this study is that it was conducted on hospitalized patients, so we can follow up and record the symptoms and duration of diarrhea more accurately than studies conducted on outpatients. However, there are some limitations to our study that need to be considered. First, this is an open-label design, which introduces bias since both parties may have preconceived notions or expectations about the treatment. Second, the sample size was small, therefore it may not detect the beneficial effectiveness between the intervention and the control groups. Finally, antidiarrheal dietary and medication use may affect the duration of diarrhea.

## Conclusions

In conclusion, it may not be necessary to use lactose-free milk routinely in Vietnamese children under 24 months with acute gastroenteritis, particularly in diarrheal children with mild or moderate dehydration and who are well-nourished, because the duration of diarrhea, weight change, treatment failure rates, and hospital stay are similar to those of children fed lactose-containing milk.
